# Convergent models of handedness and brain lateralization

**DOI:** 10.3389/fpsyg.2014.01092

**Published:** 2014-10-08

**Authors:** Robert L. Sainburg

**Affiliations:** Department of Neurology, Penn State College of Medicine, The Pennsylvania State UniversityUniversity Park, PA, USA

**Keywords:** handedness, brain lateralization, laterality, motor activity, manual asymmetry, motor lateralization, motor neurons

## Abstract

The pervasive nature of handedness across human history and cultures is a salient consequence of brain lateralization. This paper presents evidence that provides a structure for understanding the motor control processes that give rise to handedness. According to the Dynamic Dominance Model, the left hemisphere (in right handers) is proficient for processes that predict the effects of body and environmental dynamics, while the right hemisphere is proficient at impedance control processes that can minimize potential errors when faced with unexpected mechanical conditions, and can achieve accurate steady-state positions. This model can be viewed as a motor component for the paradigm of brain lateralization that has been proposed by Rogers et al. (MacNeilage et al., 2009) that is based upon evidence from a wide range of behaviors across many vertebrate species. Rogers proposed a left-hemisphere specialization for well-established patterns of behavior performed in familiar environmental conditions, and a right hemisphere specialization for responding to unforeseen environmental events. The dynamic dominance hypothesis provides a framework for understanding the biology of motor lateralization that is consistent with Roger's paradigm of brain lateralization.

## A generalized model of vertebrate brain lateralization

The division of labor between the two sides of the brain is a basic organizational feature of the vertebrate nervous system that arose in evolution even before the appearance of vertebrates (MacNeilage et al., 2009). According to the work of Rogers and colleagues, a single organizing principle might account for the large array of emotional, language, perceptual, and cognitive asymmetries that have been described across a range of vertebrate animals, including humans. They proposed that the left hemisphere has become specialized for control of well-established patterns of behavior, performed under familiar environmental circumstances, while the right hemisphere has become specialized for detecting and responding to unexpected stimuli in the environment. This elegant hypothesis was derived through seeking fundamental principles from a wide variety of experimental and natural observations of behavior. It is an example of a parsimonious principle that can account for a large range of observable behaviors, a foundation of the scientific process (Brody, 1994). Rogers further hypothesized that separating neural circuits across the hemispheres might reduce interference between potentially competing processes, thus allowing more efficient behavior. In a test of this hypothesis, Rogers and colleagues compared visual processing behaviors in groups of chicks with and without lateralized visual systems, controlled by exposing the embryo to different light regimes (Rogers et al., 2004; Vallortigara and Rogers, 2005). After hatching, the two groups of chicks were tested on a dual task, which required a normally right hemisphere process, scanning for predators, and a normally left hemisphere process, sorting food grains from pebbles. The results indicated that both groups performed each isolated task well, but only the lateralized chicks could effectively carry out the two tasks simultaneously. Thus, a single integrated behavior involving sorting food and scanning the environment is accomplished by recruiting two neural processes, across the two hemispheres. This both supports the hypothesis that neural lateralization imparts behavioral efficiency through separation of parallel neural processes, and suggests how lateralization might have contributed to natural selection in the evolutionary process. Recent research examining motor control differences between the dominant and non-dominant arms suggests that Roger's hypothesis might also explain handedness. That is, the left hemisphere (in right handers) might be specialized for controlling movements through predictive mechanisms that are most effective under consistent and stable mechanical conditions, while the right hemisphere might be specialized for impedance control, which imparts stability when mechanical conditions are unpredictable, or when stabilizing steady state position at the end of a movement.

## The dynamic dominance hypothesis provides a framework for understanding handedness within roger's hypothesis

Over the past decade, our laboratory has developed a model of motor lateralization (Sainburg, 2002, 2005; Mutha et al., 2012, 2013) that can be viewed as a motor control analog for the model of brain lateralization developed and elaborated by Rogers and colleagues. This model is based on fundamental principles of control theory that account for a range of experimental findings in different tasks and task conditions. The dynamic dominance hypothesis of motor lateralization proposes that the left hemsiphere (in right-handers) is specialized for processes that account for predictable dynamic conditions, in order to specify movements that are mechanically efficient, and have precise trajectories. In contrast, the right hemisphere (in right-handers) is specialized for impedance control mechanisms that ensure positional and velocity stabilization in the face of unpredictable mechanical events and conditions, and accuracy and stability of steady state postures. The former process assures mechanical efficiency and trajectory specificity under predictable conditions, while the latter imparts robustness under unpredictable conditions, as well as postural stability. Through studies in stroke patients with specific unilateral brain lesions, we have provided evidence that both processes contribute to control of each arm. However, the hemisphere contralateral to a given arm imparts the greatest influence to that arm's performance. In terms of Roger's hypothesis, the right hemisphere is specialized for a system that ensures stability and rapid online responses to unexpected stimuli in the internal and external environments, while the left hemisphere exploits predictive processes to assure trajectory precision and mechanical efficiency when conditions are consistent and predictable.

## Hybridization of predictive and impedance mechanisms allows efficient and robust control of movements

Energy conservation has clearly played a significant role in the process of human evolution, contributing to our tendency to exploit coordination patterns that are energy efficient (Alexander, 1997; Nishii and Taniai, 2009). Predictive mechanisms can be used in order to minimize costs, such as energy and smoothness, when environmental conditions are predictable. Thus, optimality is an important principle for predictive control (Todorov, 2004). However, because environmental conditions are often unpredictable, impedance control through modulation of feedback gains is also an important component of biological movements (Scott, 2004; Mutha et al., 2008; Omrani et al., 2013). Indeed, from a mechanical perspective, the world can be very unstable and unpredictable. For example, inertial interactions while riding in a vehicle and holding or reaching for a cup of coffee can be quite large when changes in acceleration are not anticipated. Similarly, slicing an irregular shaped piece of fruit or vegetable can be unstable because it can slip or rock with force components applied by a knife. It should also be stressed that one's own motor commands can introduce unanticipated errors in intended movements, due to errors in prediction, and noise in central processes that might include erroneous sensory estimates (Faisal and Wolpert, 2009). Thus, in addition to predictive mechanisms that can produce smooth and efficient coordination patterns, impedance mechanisms can assure stability in the face of unexpected external and internal conditions, and can assure steady state positions at the end of motion.

Predictive control mechanisms can be used to optimize a combination of kinematic and dynamic costs of movement (Hogan and Sternad, 2009; Yadav and Sainburg, 2011). Examples of component costs that have been proposed in the literature include Movement Smoothness, Mean Squared Torque, Peak Work, Muscle Energy and Final Position Variability (Osu et al., 1997; Kawato and Wolpert, 1998; Kawato, 1999; Harris and Wolpert, 2006). However, predictive control based on such optimization principles, whether implemented through open loop or optimal feedback control schemes (Todorov, 2005), is not robust to unanticipated changes in task conditions. In addition, achieving stable final positions through such mechanisms can be sensitive to internally generated prediction errors and neural noise. In fact, in a recent series of experiments, Scheidt and Ghez (Ghez et al., 2007; Scheidt and Ghez, 2007) demonstrated independent mechanisms for controlling limb trajectory and final position during reaching movements. According to their findings, trajectory control was generated largely by predictive mechanisms, and final position stability was achieved largely through mechanisms similar to impedance control.

How can impedance control counter unanticipated perturbations and stabilize final positions? Mechanical impedance includes 3 components that vary with acceleration, velocity, and position. While the first is dependent on inertia, and cannot actively be modulated, the effective stiffness-like and viscous-like behavior of the limb can be neurally modulated (Shadmehr and Arbib, 1992). The mechanisms through which impedance modulation can occur include muscle co-activation (Gomi and Kawato, 1997; Burdet et al., 2001; Osu et al., 2009), as well as modulation of proprioceptive reflex gains and thresholds (Mutha et al., 2008; Pruszynski et al., 2011). It has previously been demonstrated that impedance mechanisms can provide stability of the trajectory and final position during the initial phases of motor learning (Takahashi et al., 2001), or when environmental conditions are unstable or unpredictable (Milner and Franklin, 2005; Burdet et al., 2006). Schabowsky et al. (2007) and Duff and Sainburg (2007) have shown that the non-dominant arm tends to rely on impedance control for adaptation, even when conditions are predictable, whereas, the dominant arm tends to rely on predictive mechanisms to a greater extent. However, impedance control mechanisms cannot be used to optimize factors such as energy expenditure, and thus can result in high energetic costs. This is consistent with the finding that the non-dominant arm, which relies on such control, tends to perform movements with higher energetic cost than the dominant arm (Bagesteiro and Sainburg, 2002) Thus, each control scheme offers advantages, which can counter the disadvantages of the alternate control scheme.

**Figure 1:**
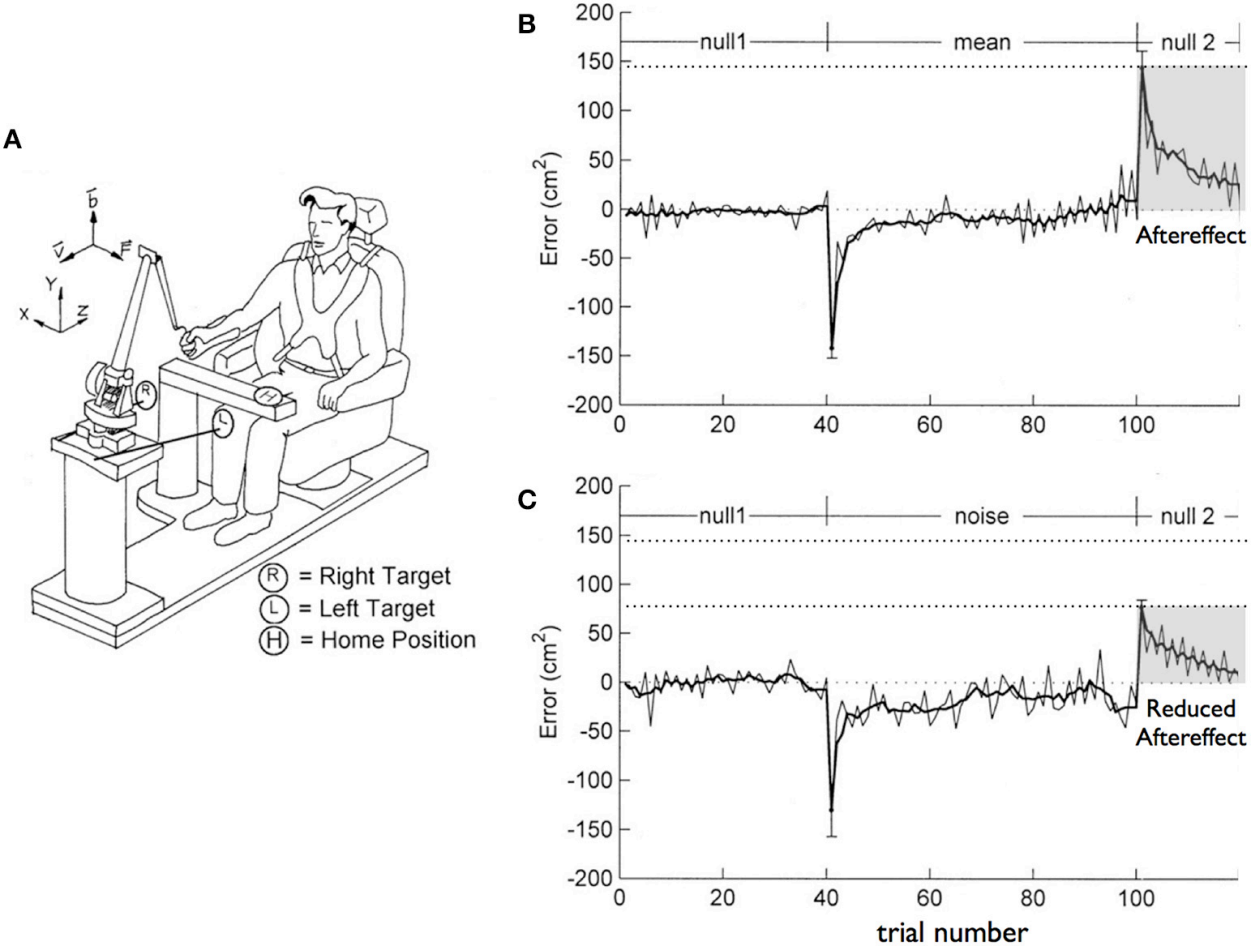
**(A)** Experimental Setup. Subjects held a robotic manipulandum while reaching to targets to the left and right of midline. **(B)** Perpendicular errors during the course of the session in which subjects experienced the consistent field. **(C)** Perpendicular errors during the course of the session in which subjects experienced the inconsistent (noisy) field (from Takahashi et al., 2001).

The hybridization of predictive and impedance control mechanisms for smooth and energetically efficient movements that can resist unpredictable mechanical interactions has previously been well-established. For example, Takahashi et al. (2001) exposed subjects to two alternative force fields imposed by a robotic manipulandum (see Figure 1A), while they reached toward targets with the dominant arm. The force fields were proportional to velocity and directed perpendicular to the targeted movements, tending to impose perpendicular deviations in the movement paths. Subjects were either exposed to a consistent field, or a field that varied in magnitude from trial to trial, but had the same mean amplitude as the consistent field. When initially exposed to the consistent field, subjects showed large errors in the direction of the field (Figure 1B, negative peak), yet over practice were able to eliminate these errors. When the field was removed following adaptation, aftereffects were directed in the opposite direction to the initial errors (Figure 1B, positive peak). Such aftereffects have previously been well-characterized, and are thought to represent predictive mechanisms that account for the previously applied field (Lackner and DiZio, 1998; Wang et al., 2001; Hwang and Shadmehr, 2005). In this study, when subjects were exposed to the inconsistent amplitude field, they also adapted (Figure 1C, positive peak). However, following adaptation, the amplitude of the aftereffects were substantially smaller than that following the consistent field. These results indicated the addition of impedance mechanisms that helped reduce the amplitude of errors. Trial-to-trial analysis revealed that impedance mechanisms were used in combination with predictive control to reduce the effects of unanticipated variations in force. This study, as well as a number of related adaptation studies (Ghez et al., 2007; Scheidt and Ghez, 2007; Yadav and Sainburg, 2014), demonstrated the use of a hybrid control strategy, exploiting both predictive and impedance mechanisms for efficient and robust coordination of arm movements.

## Lateralization of predictive control mechanisms

**Figure 2:**
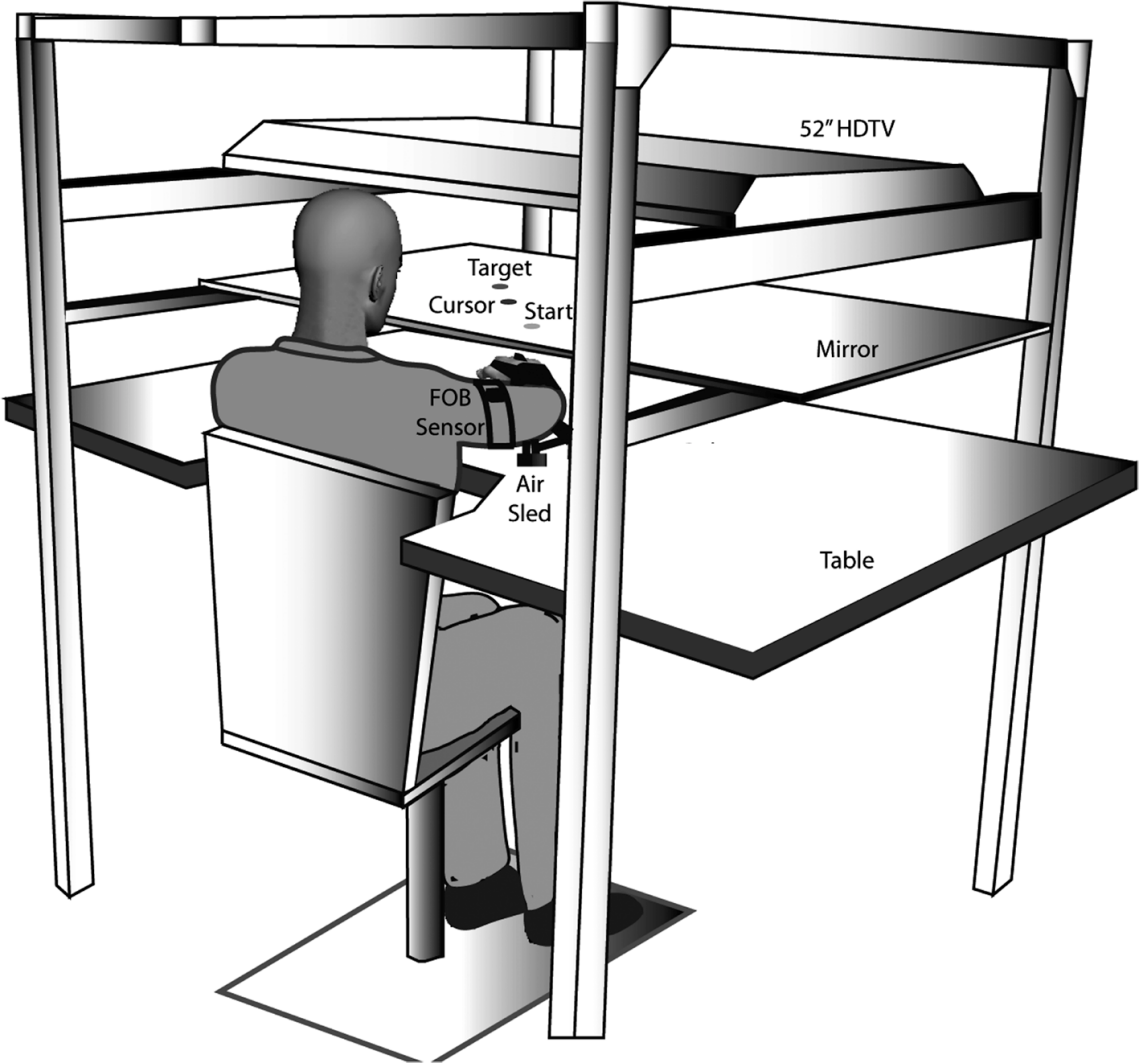
**Experimental set-up: Subjects sat facing a table with their arm supported in an air-sled over the horizontal surface by an air-jet system**. An LCD screen was positioned above the mirror, which reflected a 2-D virtual reality environment, in which a start position and target were presented (from Yadav and Sainburg, 2014).

There has been substantial evidence that the two control mechanisms described above are specialized in different cerebral hemispheres, imparting different control characteristics to each arm. In a number of previous studies, we have characterized dominant arm advantages for predictive control during reaching movements (Sainburg and Kalakanis, 2000; Bagesteiro and Sainburg, 2002; Sainburg, 2002, 2005; Duff and Sainburg, 2007; Wang and Sainburg, 2007; Shabbott and Sainburg, 2008; Tomlinson and Sainburg, 2012; Mutha et al., 2013; Yadav and Sainburg, 2014). Figure 2 shows the general experimental set up for our reaching studies. Subjects are seated in front of a table, while an air jet system allows the arms to glide over the surface, thus minimizing the effects of both friction and gravity. A virtual reality interface is projected on a mirror, placed horizontally above the arm, and under a 55″ HDTV monitor. This allows projection of a virtual or veridical location for a cursor, that represents the subjects' hand position.

**Figure 3:**
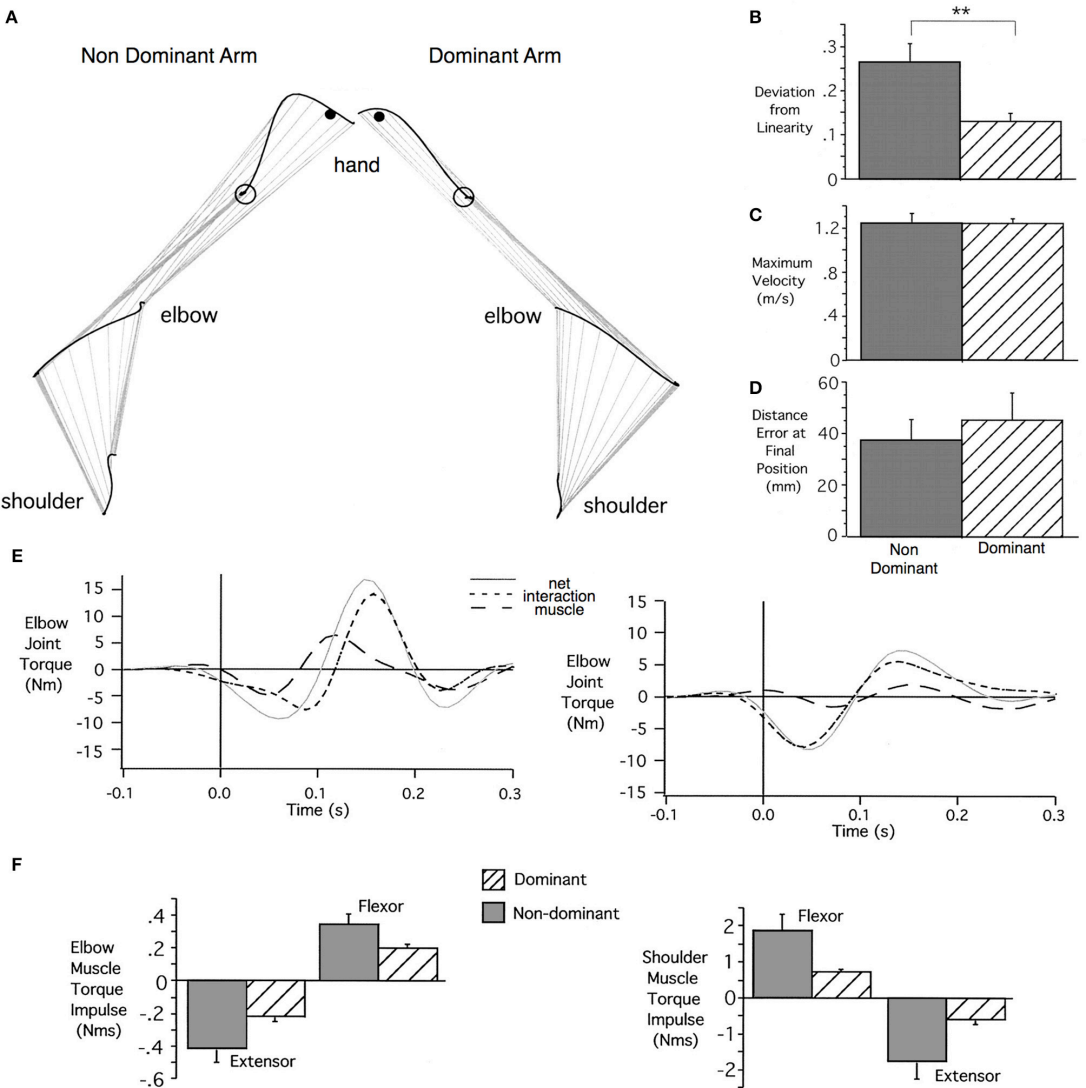
**(A)** Shoulder, elbow, and hand trajectories from typical left (non-dominant) and right (dominant) hand arm movements toward a medial target. **(B–D)** Mean's ± *SE* for Deviation from Linearity **(B)**, Maximum Velocity **(C)**, and Distance Errors **(D)** for all movements across all subjects. **(E)** Elbow joint torque profiles include muscle torque, interaction torque, and net torque. **(F)** Group mean's ± *SE* for integrated flexor (positive) and extensor (negative) elbow and shoulder joint muscle torques (from Bagesteiro and Sainburg, 2002).

Figure 3 shows examples of left and right arm horizontal plane reaching movements, performed rapidly without visual feedback, for a typical right-handed individual (Bagesteiro and Sainburg, 2002). As reflected by the graphs at the right, when dominant and non-dominant arm movements are matched for speed, dominant trajectories are substantially straighter, but tend to have slightly larger final position errors than non-dominant arm movements. In contrast, non-dominant trajectories tend to be deviated away from the target position, curving back toward the target at the end of motion. Figure 3 (middle) shows the elbow joint kinetics associated with these two movements. Most notable is the fact that the computed muscle torque profile, reflecting muscle actions, remains near zero throughout the dominant arm movement. Nevertheless, the elbow achieves substantial net torque because the dominant controller efficiently exploits the interaction torque (dashed) that results from shoulder motion to drive the elbow joint into extension. In contrast, the non-dominant arm generates excessive elbow muscle torque that combines with interaction torque to deviate the hand path laterally. The result is the generation of a directionally inaccurate movement that requires substantially greater muscle torque at both joints to generate the same speed movement to the target. As reflected by the bar plots in Figure 3 (bottom), dominant arm movements used substantially less integrated shoulder and elbow muscle torque to achieve comparable movement distances, speeds, and accuracies. This supports the idea that dominant arm movements are characterized by a control strategy that takes advantage of non-muscular forces. Nevertheless, non-dominant movements tend to achieve equal or slightly better final position accuracies, probably related to impedance control that can achieve accurate steady state positions. We have corroborated these findings in vertical reaching movements, performed without support (Tomlinson and Sainburg, 2012), and in left-handers (Przybyla et al., 2012). In related studies, we have confirmed that both energetic costs, and normalized muscle activities are higher in non-dominant arm reaching movements, while final position errors tend to be lower (Sainburg and Kalakanis, 2000; Bagesteiro and Sainburg, 2003).

**Figure 4:**
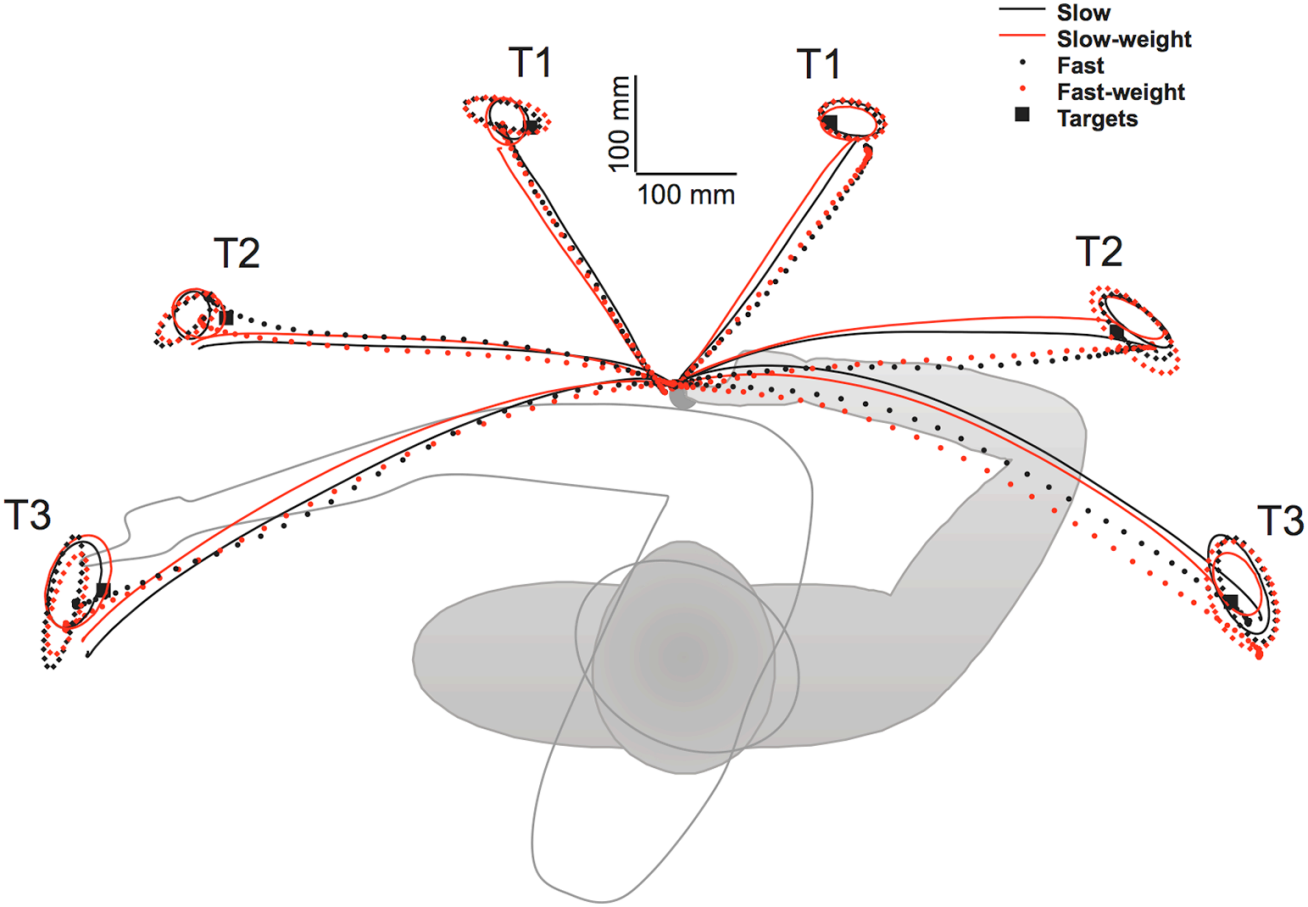
**Experimental set-up and averaged trajectories for turn and reach movements made with the left (right side of workspace) and right (left side of workspace) hands of right-handers under both speed (slow, fast) and weight (weight, no weight) conditions**. Ellipses depict 95% confidence intervals for end point distributions under each condition (from Pigeon et al., 2013).

Similar findings have been reported for different types of movements from other research groups. For example Pigeon et al. (2013) reported interlimb differences in coordinated turn-and-reach movements performed while standing. As shown in Figure 4, right handed subjects reached to 3 targets on the left of midline with the right arm, and 3 targets on the right of midline with the left arm. Movements were performed at two speeds (slow and fast) and under two loading conditions (1 lb weight, no weight). Due to the required trunk rotation, substantial Coriolis forces acted perpendicular to the target direction. As reflected by the paths in Figure 4, dominant arm movements were straighter and were minimally affected by the speed and weight conditions. In contrast, non-dominant arm movements were deviated laterally, more curved, and varied substantially with mass and speed conditions. Thus, the dominant arm was able to take account of the non-muscular Coriolis forces generated by trunk rotation, whereas non-dominant arm movements were substantially deviated by these interactions. Nevertheless, non-dominant arm movements curved back toward the targets at the end of motion, and were slightly more accurate with respect to radial errors at the final steady-state position.

Hore and colleagues extended these findings to overarm throwing movements. They conducted a series of studies of overarm throwing in the dominant and non-dominant arms, demonstrating that dominant arm movements take advantage of the whipping actions of interaction torques to generate accurate and high velocity motions of the hand at ball release (Hore et al., 1996, 1999, 2005; Debicki et al., 2004, 2011). In fact, for the dominant arm, coordination patterns between the joints was qualitatively different for slow and fast throws, as subjects incorporated non-muscular interaction torques into the faster motions. In contrast, the non-dominant arm did not exploit these interactions, but instead exhibited the same intersegmental coordination patterns for both fast and slow movements. Thus, the greater-skill of the dominant arm was associated with the exploitation of non-muscular intersegmental interaction torques for rapid throwing motions. Heuer further extended this line of research to include tapping-like movements of the fingers (Heuer, 2007). During rapid finger oscillations, the dominant hand coordination strategy was shown to exploit non-muscular forces, while the non-dominant arm used excessive muscle co-contraction to impede the action of such forces. This resulted in greater efficiency and temporal consistency in motions of the dominant arm.

Taken together, these studies demonstrate that the dominant system is able to account for and exploit limb and task dynamics to make well-directed, smooth, and energetically efficient movements. Non-dominant movements tend to be less-efficient, and are often perturbed by non-muscular interactions. These findings lead to the conclusion that the left hemisphere (in right handers) control system is specialized for coordinating limb and task dynamics, a process that has been shown to rely on feedforward use of vision and proprioception in predictive control processes (Ghez et al., 1990, 1994, 1995; Sainburg et al., 1993; Gordon et al., 1994; Ghez and Sainburg, 1995).

## Lateralization of impedance control mechanisms

As elaborated above, even though the non-dominant arm tends to make less energetically efficient movements that are deviated by non-muscular forces, the final steady state position accuracy tends to be as good or better than that of the dominant arm. This likely reflects the exploitation of positional impedance mechanisms that can specify stiffness about equilibrium postures (Foisy and Feldman, 2006). In fact, a variety of studies have converged to suggest that the non-dominant arm exploits impedance mechanisms to generate accurate and stable arm movements. Studies of non-dominant arm adaptation to consistent viscous (Schabowsky et al., 2007) and inertial (Duff and Sainburg, 2007) loads have shown that adaptation occurs largely by impeding the trajectory deviations imposed by the force fields, rather than by specifically countering the fields through predictive mechanisms. While the non-dominant arm adapts to the applied force fields, aftereffects, reflecting predictive control mechanisms, tend to be small and inconsistent. In contrast, dominant arm adaptation to the same fields is characterized by large aftereffects that mirror the initial errors introduced by exposure to the forces. These findings support the hypothesis that impedance mechanisms are exploited to a greater extent by the non-dominant arm during adaptation to novel force fields. It has also been shown that the non-dominant arm responds to unexpected inertial loading with greater final position accuracy than the dominant arm (Bagesteiro and Sainburg, 2003). These findings suggest that the impedance control mechanisms employed for non-dominant arm control might be based, to some extent, on proprioceptive feedback loops. This may, in turn, be related to findings that the non-dominant arm shows an advantage in proprioceptive matching tasks (Goble et al., 2006, 2009; Goble and Brown, 2008a). In addition, the non-dominant arm tends to achieve more accurate final positions, when reaching movements are made without visual feedback of the hand, toward a large number of targets throughout the workspace (Oyama, 2012; Przybyla et al., 2013). Thus, the non-dominant arm exploits impedance control mechanisms to a greater extent than predictive mechanisms when adapting to novel dynamic conditions, and tends to achieve more accurate steady state positions, when confronted with unexpected inertial loads, or requirements for achieving steady state positions without the aid of visual feedback. Together, these findings provide support to the idea that the right hemisphere (in right handers) controller relies on impedance control mechanisms that exploit proprioceptive feedback loops to specify steady state limb configurations.

**Figure 5:**
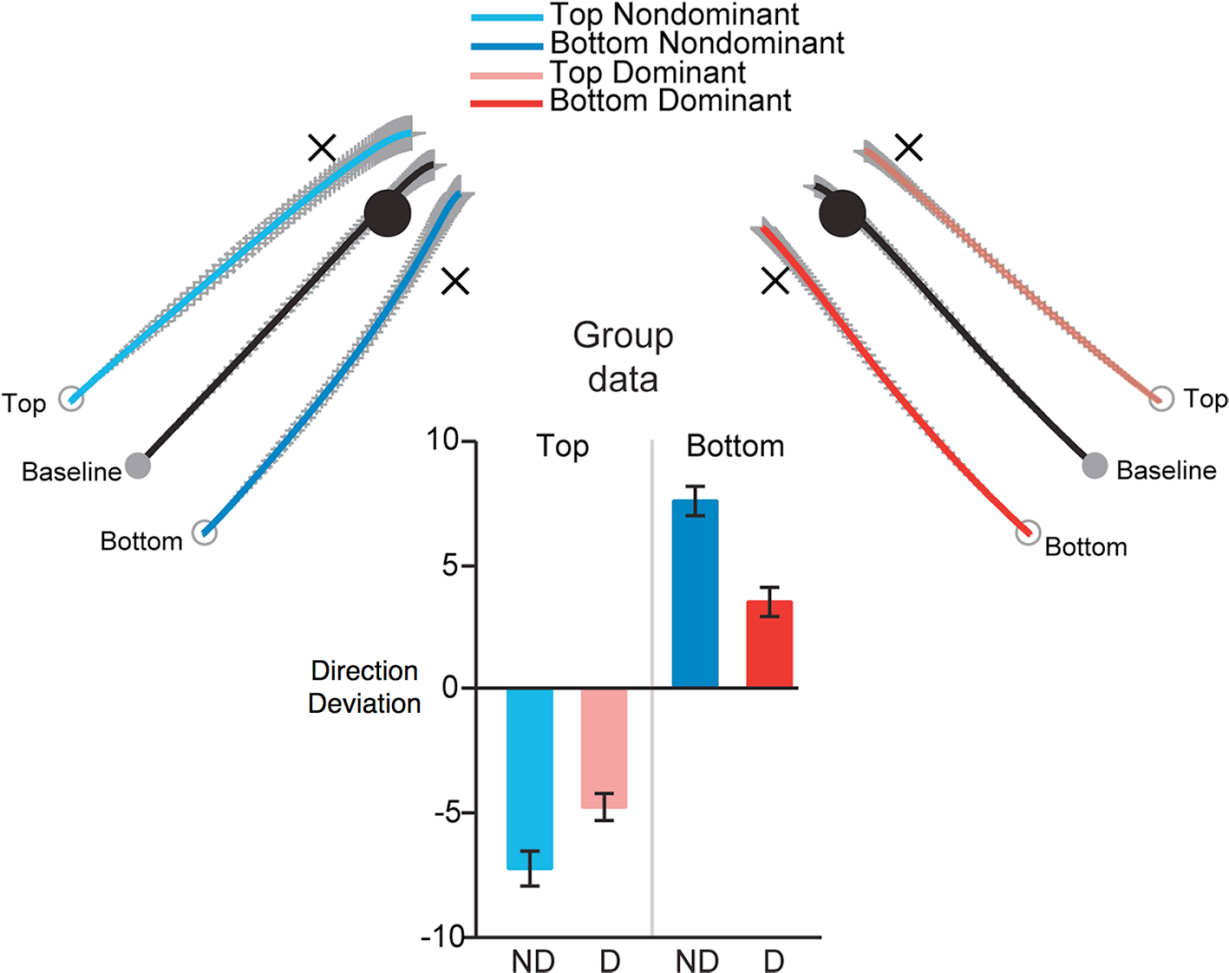
**Groups means for trajectories (normalized and averaged across subjects) made toward targets under baseline (black) and from displaced initial positions, for dominant (red) and non-dominant (blue) arms**. Bar plots (right) show mean ± *SE* for direction error, measured as the difference in the direction of displaced and baseline movements, measured at final position (from Mutha et al., 2013).

We designed an experiment to specifically address whether the non-dominant arm might optimize positional stability by specifying impedance around equilibrium positions, while dominant arm movements rely on predictive mechanisms that specify movement trajectories (Mutha et al., 2013). In a targeted-reaching experiment, we covertly and occasionally shifted the starting position of the hand, perpendicular to the direction of the target. We hypothesized that non-dominant control is specialized for achieving stable postures by specifying impedance around “equilibrium” positions. For goal-directed arm movements, this control mechanism should specify a “threshold” or “referent” configuration for the arm, similar to that proposed by the equilibrium point hypothesis (Feldman et al., 1995, 2011; Foisy and Feldman, 2006). Consistent with this, the non-dominant arm often shows better accuracy and precision in achieving a desired spatial position, particularly when an ongoing movement is perturbed (Bagesteiro and Sainburg, 2003; Duff and Sainburg, 2007; Przybyla et al., 2013). We, thus predicted that under conditions in which the starting position of the hand is shifted perpendicular to the target direction, non-dominant arm movements should reproduce the final equilibrium position of the baseline movements, whereas the dominant arm trajectory should parallel that of the baseline movements. The results of this study are represented in Figure 5. Dominant arm movements (Right) largely paralleled baseline movements and thus had smaller direction differences (direction errors-bar plot), than baseline movements. In contrast, non-dominant arm movements converged to the baseline final position and had larger direction differences than baseline movements. However, it is important to note that non-dominant arm movements did not completely converge onto the baseline target. The angular deviation was about 60% of that required to land the arm exactly on that target. Similarly, dominant arm movements were not completely parallel to baseline trajectories, especially for the medial displacements. These results suggest that each arm uses a predominant strategy, but not an exclusive control strategy. Thus, the dominant arm relies mostly on predictive control, but also employs impedance mechanisms, and vice versa for the non-dominant arm. This evidence provides support for hybrid control of each arm.

## Computational hybrid-control simulation

**Figure 6:**
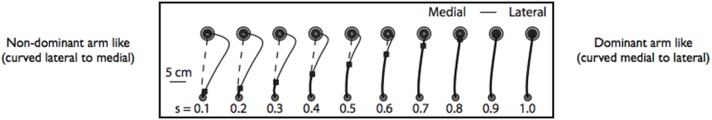
**Simulated trajectories for different switch times between predictive and impedance controller**. Dashed line shows pure optimal predictive controller. Early switch times **(Left)** are controlled almost entirely by the impedance control algorithm, while late switch times **(Right)** are almost entirely controlled through optimal predictive control (form Yadav and Sainburg, 2014).

The evidence provided above suggests that hybrid control might be the foundation for handedness. In order to examine the plausibility of our hypothesized hybrid control scheme, we developed a computational simulation that combined predictive control of limb dynamics with impedance control mechanisms, in a serial control scheme, to characterize the differences between the trajectories of dominant and non-dominant arm movements. In this simulation, the movements of both the arms were initiated using predictive control mechanisms, and terminated using impedance mechanisms (Yadav and Sainburg, 2011, 2014, Neuroscience). We reasoned that the different coordination patterns between the limbs might reflect the degree to which the movement depends on each mechanism during its course, which we characterized in this simulation as the time that control switched from predictive to impedance mechanisms. Four parameters were used to characterize predictive control, four parameters for impedance control, and a 9th parameter described the instant of switch between the two modes of control. We predicted an early switch to impedance control for the non-dominant arm, but a late switch, near the end of motion, for the dominant arm. Figure 6 shows the results of this simulation for different switch times during the course of a typical movement. Note that these trajectories are shown in a right hand coordinate system. For early switches to impedance control (left side of Figure 6), movements were deviated laterally, and curved back toward the target at the end of motion, while late switches (right side of Figure 6) are fairly straight. These different trajectories are very similar to the right and left arm paths shown in Figure 3 for rapid, horizontal plane reaching movements. In fact, when we optimally fit our model to subjects' movements, the more curved trajectories of the non-dominant arm were best characterized by a significantly earlier switch to impedance mechanisms than when the model was fit to dominant arm movements. The trajectories of the dominant arm were best fit, when the switch to impedance mechanisms occurred late in the deceleration phase of motion. This simulation provided confirmation that hybrid control using both impedance and predictive control mechanisms is plausible and might explain the trajectory differences of dominant and non-dominant arm reaching movements.

## The effects of hybrid control on motor performance and adaptation

**Figure 7:**
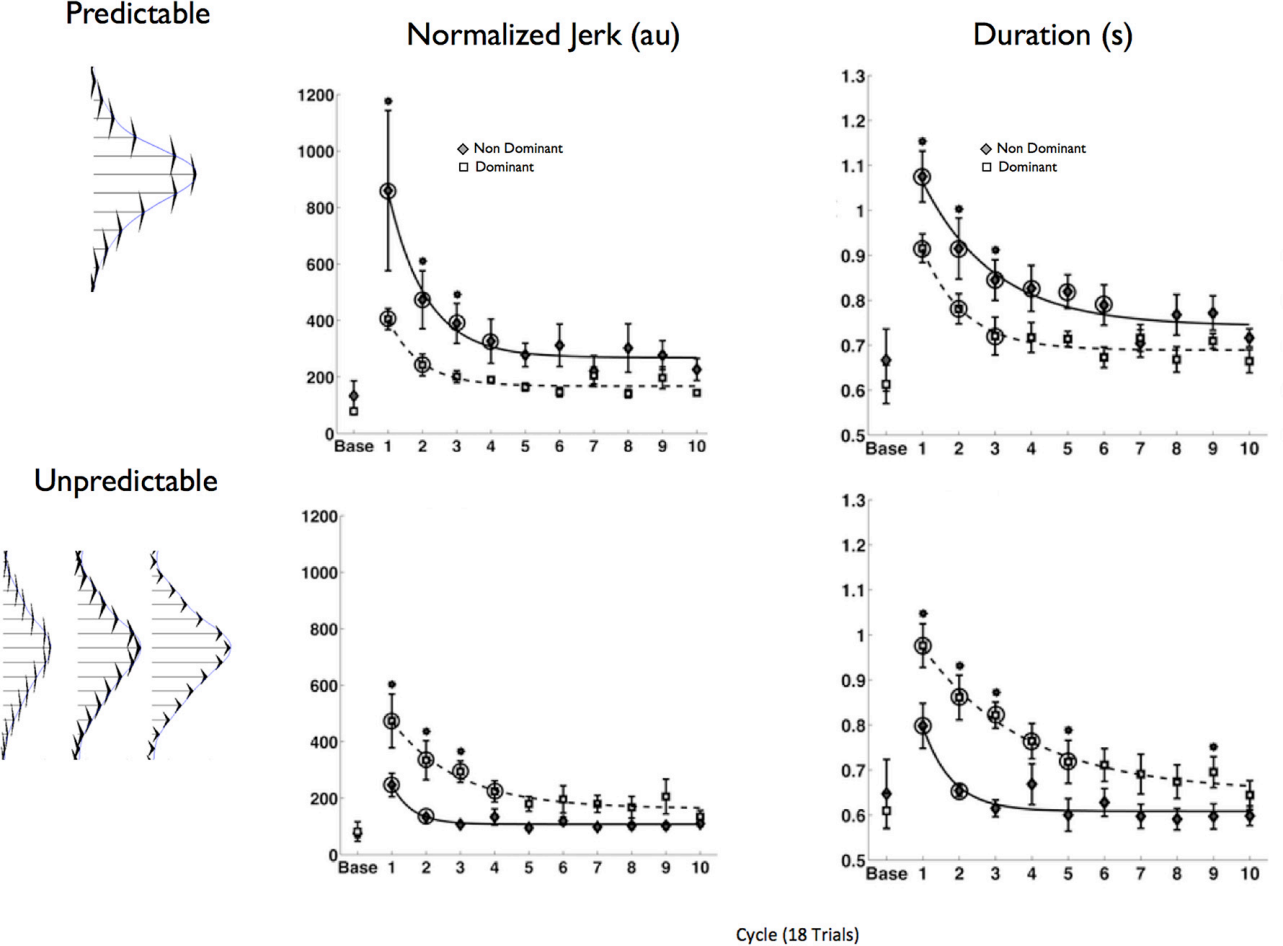
**Force field structure is shown at right: fields were generated perpendicular to the direction of the target, and varied with either the square of velocity (predictable field) or linearly with velocity (unpredictable)**. Group mean ± *SE* for mean squared jerk **(Left)** and movement duration **(Right)** are shown across all 180 movements (18 cycles). Baseline performance is shown at the left of each plot (form Yadav and Sainburg, 2014).

In a direct test of the hypothesis that the non-dominant arm exploits predominantly impedance mechanisms, while the dominant arm exploits predominantly predictive mechanisms for control, we designed a study (Yadav and Sainburg, 2014) that employed a similar paradigm to that introduced by Takahashi et al. (2001). However, rather than exposing only the dominant arm to a predictable and unpredictable field, we exposed each arm to the both fields. Each force field was imposed by a robotic manipulandum attached to the arm support. The field that was designed to advantage the predictive controller had a consistent magnitude between trials, that varied with the square of hand velocity. The field designed to advantage the impedance controller had an inconsistent magnitude between trials that varied linearly with hand velocity. Because the velocity-square field did not change the form of the equations of motion for the reaching arm, we reasoned that a forward dynamic-type controller should perform well in this field, while control of linear damping and stiffness terms should be less effective. In contrast, the unpredictable linear field should be most compatible with impedance control, but incompatible with predictive dynamics control. Our hypothesis predicted an arm X field interaction, such that the dominant arm should perform best within the consistent field, and the non-dominant arm in the inconsistent field. Figure 7 shows the results of this experiment, quantified by mean squared jerk, a measure that varies inversely with movement smoothness (Left), and movement duration (right). Both measures of performance showed a hand X field interaction, such that dominant arm movements were performed smoother and faster within the predictable field, while non-dominant arm movements were performed smoother and faster within the unpredictable field. These findings corroborated our hypothesis that motor lateralization might reflect asymmetries in specific motor control mechanisms associated with predictive control of limb and task dynamics, and control of limb impedance.

## Is hybrid control of limb dynamics and limb impedance based on hemispheric specializations?

Previous studies have demonstrated that following unilateral stroke, motor impairment occurs both contralateral, as well as ipsilateral to the lesion (Wyke, 1967; Winstein and Pohl, 1995; Hermsdorfer et al., 1999b; Swinnen et al., 2002; Haaland et al., 2004, 2009; Yarosh et al., 2004; Wetter et al., 2005; Sainburg and Duff, 2006; Schaefer et al., 2007, 2009a; Chestnut and Haaland, 2008). Although ipsilesional impairments can be functionally limiting, they can also provide important insight into the role of the ipsilateral hemisphere in controlling movement. Specifically, the lateralization of specific motor control mechanisms can be examined, given that unilateral arm movements are thought to recruit processes in both hemispheres. Our hypothesis of hybrid control has two important predictions for unilateral brain lesions that affect sensorimotor function: First, because we hypothesize that both hemispheres contribute different mechanisms to each arm, unilateral hemisphere lesions should produce hemisphere specific deficits in the ipsilesional arm of stroke patients. Therefore, control of the ipsilesional arm should reflect a greater influence from contributions of the contralesional controller, when compared with the same arm of age matched control subjects. We limited our analysis to patients with right handedness, given the lack of normative data on lefties, and because of restrictions in recruitment. We initially focused our study on patients with significant hemiparesis, on the contralesional side of the body.

**Figure 8:**
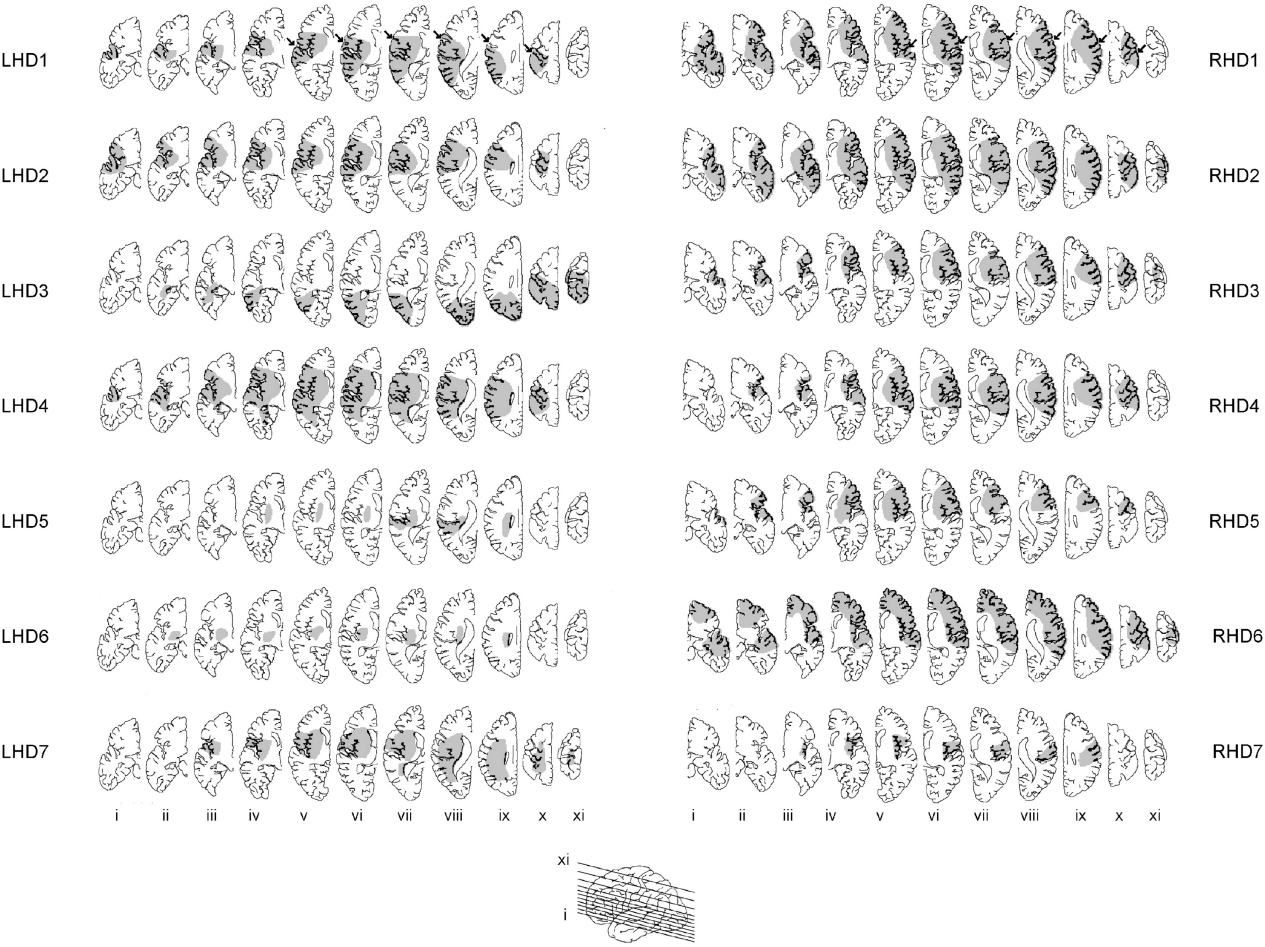
**Lesion locations were traced on 11 axial slices (see insert for slice level) from MRI or CT scans for each LHD (1–7) and RHD (1–7) patient**. Slices are displayed left-to-right from inferior to superior (i–xi) for both groups of patients. Arrows in top row indicate location of central sulcus (from Schaefer et al., 2009a).

**Figure 9:**
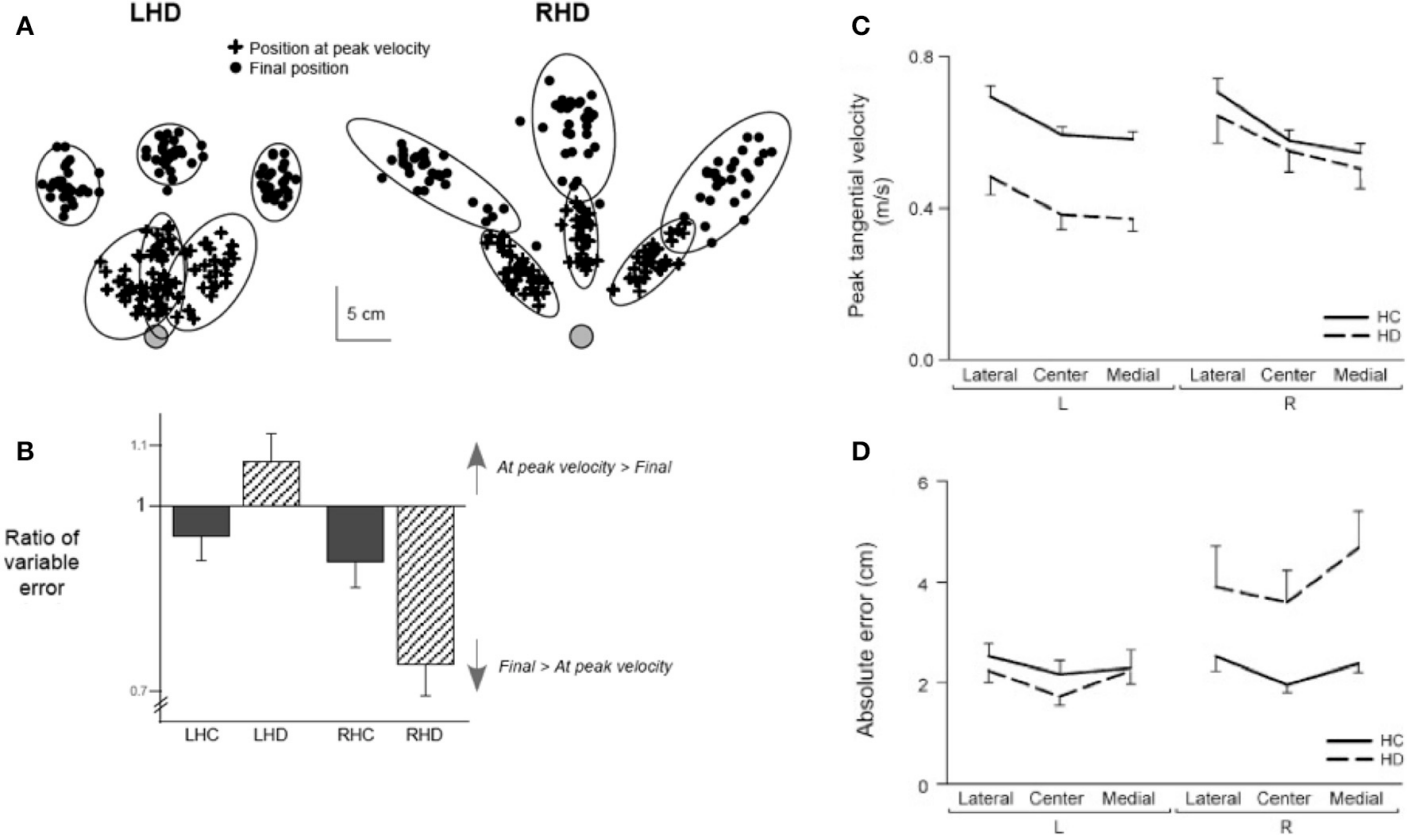
**(A)** Sample positional variation plots, with 95% confidence intervals, shown by ellipse, for an example LHD patient and RHD patient. **(B)** Group data shows ratio of positional variation at peak velocity divided by positional variation at the end of movement for LHD, RHD, left hemisphere control group (LHC), and right hemisphere control group (RHC). **(C)** Mean ± *SE* for peak velocity and **(D)** absolute final position error for all four groups (LHD, RHD, LHC, RHC) for movements to each target (from Schaefer et al., 2009a).

The main purpose of this study was to examine whether our dynamic dominance model of motor lateralization could predict hemisphere specific motor deficits in stroke patients (Schaefer et al., 2009a). Chronic stroke patients with either left or right hemisphere damage (LHD or RHD) used their ipsilesional arm, and the control subjects used either their left or right arm (LHC or RHC), to perform targeted reaching movements in different directions within the workspace ipsilateral to their reaching arm. We used structural MRI images to quantify the location and volume of each subjects' lesion, in order to match lesion characteristics between our LHD and RHD groups (see Figure 8). The results of the study are depicted in Figure 9, which shows variability in performance at two points in the movement, at peak velocity, or at the final position. The ellipses reflect 95% confidence intervals around the cloud of hand path points for representative patients with left and right hemisphere damaged patients. LHD patients had greater variabilities early in movement and significantly greater initial direction errors and trajectory curvatures than both age matched control subjects (LHC) and RHD patients. In contrast, RHD patients showed lower initial trajectory variabilities and trajectory deviations, but greater final position variances and errors than both their control group and LHD patients. Left hemsiphere damage produced deficits in controlling the ipsilesional arm trajectory, whereas the RHD group showed deficits in ipsilesional final position accuracy. These results extended our findings in asymmetrical control of each limb in healthy subjects to the cerebral hemispheres: We showed that each hemisphere contributes different control mechanisms to the ipsilesional arm. While the existence of spasticity and paresis precluded the examination of contralesional arm function in this group of patients, we later extended these findings to the contralesional arm in patients with very mild paresis (Mani et al., 2013). In addition, studies examining the role of each hemisphere in visuomotor adaptation paradigms have also supported the hypothesis that each hemisphere contributes different control processes to each arm: We found that LHD interfered with adaptation of initial direction, but not with the ability to adapt the final position of the ipsilesional arm. In contrast, RHD interfered with online corrections to the final position during the course of adaptation. These findings support our hypothesis that the control of trajectory and steady-state position may be lateralized to the left and right hemispheres, respectively (Schaefer et al., 2009b). Thus, substantial evidence in stroke patients supports the proposition that each hemisphere contributes hemisphere specific mechanisms to control of each arm.

## Is arm selection related to motor control asymmetries?

Handedness is most often measured by questionnaires that assess an individual's preference for using a particular hand to perform a variety of tasks. While such assessments have proved reliable, they do not address the underlying neurobehavioral processes that give rise to the choice of which hand to use. The result is that preference measures can give quite different results under different task conditions (Stoloff et al., 2011; Habagishi et al., 2014). In fact, Coelho and colleagues have shown that choice of hand is subordinated to other task constraints, such as the maneuverability of the hand, following retrieval of an object, when the two are set up in a competing paradigm (Coelho et al., 2014). Thus, it is clear that arm selection is not simply a reflection of lateralization of motor performance, but results from an interaction between control asymmetries and task requirements.

In order to better understand this interaction, we recently conducted a series of studies based on the hypothesis that an individual's choice of which hand to use for a given task should result from an interaction between the underlying control asymmetries with task conditions (Przybyla et al., 2013; Mani et al., 2014). We tested this hypothesis by manipulating two factors in targeted reaching movements that differentially affect limb performance: Region of workspace, and visual feedback condition. The first manipulation modified the geometric and dynamic requirements of the task for each arm across 32 targets that occupied a large range of the reachable horizontal workspace in front of the subject. The second variable, visual feedback condition, modified the sensorimotor performance asymmetries. Previous evidence indicated that the non-dominant left arm often shows equal or greater accuracy compared to the dominant right arm, when performing reaching movements in the absence of visual feedback, but worse accuracy when vision is available (Guiard et al., 1983; Carson et al., 1990; Imanaka et al., 1995; Lenhard and Hoffmann, 2007; Goble and Brown, 2008b). This is likely related to the fact that dominant arm predictive processes are dependent upon vision for updating, and degrade in the absence of visual information (Ghez et al., 1994). However, non-dominant arm control appears to be less dependent upon visual information, which is consistent with the idea that non-dominant control relies more completely on proprioceptive information (Bagesteiro and Sainburg, 2002; Goble and Brown, 2010). Thus, we reasoned that manipulating visual feedback allowed us to experimentally vary the relative performance advantages between the arms, providing an advantage of the non-dominant arm under no-vision conditions, and to the dominant arm under vision conditions.

Our results confirmed these predictions, demonstrating a substantial advantage for the non-dominant arm when performing in the absence of visual feedback, and for the dominant arm with visual feedback. In addition, removing visual feedback increased the choice to use the non-dominant arm to reach toward targets near midline, an effect that was enhanced for targets requiring larger movement amplitudes. These results showed that limb choice is an interactive process, based on current sensorimotor conditions, in the context of a given task. Most importantly, these results support the view that limb selection emerges from the underlying control processes that confer advantages to each limb under specific task conditions. While these underlying neural processes appear to be constant, they can result in either limb experiencing performance advantages, depending on task conditions. Thus, limb selection should be viewed as an emergent phenomenon that results from the interaction between lateralization of basic motor control processes with current task conditions. For this reason, limb selection should not be viewed as a primary factor in either measuring or in defining motor lateralization.

## Summary and conclusions

This paper presented evidence for the Dynamic Dominance Model of motor lateralization that proposes a left hemisphere (in right-handers) specialization for processes that predict the effects of limb and task dynamics, given consistent mechanical conditions, and a right hemisphere specialization for impedance control mechanisms that can minimize potential errors when faced with unexpected mechanical events. This model forms a motor specific component to the broader paradigm of brain lateralization that has been proposed by Rogers et al. (MacNeilage et al., 2009). Roger's model attributes specialization of the left-hemisphere of the vertebrate brain to well-established patterns of behavior performed in familiar environmental conditions, while the right hemisphere is seen as specialized for responding to unforeseen environmental events. The dynamic dominance model of motor lateralization seems to form the motor specific analog to these specializations. The fit between these two models is particularly impressive, given that the research was derived independently. Roger's model was developed by seeking fundamental principles that could explain a wide variety of experimental and natural observations of behavior across a range of vertebrate species. The dynamic dominance hypothesis was independently developed by seeking an organizational principle that could account for motor asymmetries in humans, and hemisphere specific motor deficits in patients with unilateral brain lesions. Both hypotheses seem to converge in supporting a global framework for understanding the biology of motor lateralization.

Rogers model presents an elegant organizing principle that can encompass a large array of emotional, language, perceptual, and cognitive asymmetries across a spectrum of vertebrate species. However, it remained unclear how exactly handedness might fit into this model. Certainly, it is well-established that humans and certain species of non-human primates (Hopkins and Bard, 1993; Hopkins and Bennett, 1994; Hopkins and Cantalupo, 2004; Hopkins and Russell, 2004; Hopkins et al., 2005) prefer the right hand for performance of tasks using tools, for overhand throwing, and other skilled behaviors. Further, these tasks could be considered as best performed in predictable environmental circumstances. However, the fit between these observations of arm preference and the model expressed by Rogers has not been clear, nor has the role of the non-dominant arm within this scheme been elaborated. Over the past few decades, studies of motor coordination in healthy individuals and of hemisphere specific deficits in stroke patients have provided evidence for an explanation of handedness that is based on fundamental motor control principles. The role that each mechanism contributes to control depends on the predictability and consistency of the mechanical environment. Impedance control processes take precedence under unpredictable and unstable mechanical environments, while predictive processes prevail when environmental conditions are consistent and predictable. Right hemisphere processes that impart impedance control to the limbs lead to robust, but inefficient behavior, whereas left hemisphere processes that provide for predictive control can lead to energetically efficient coordination patterns. This paper has reviewed substantial evidence that these two aspects of control are specialized in different cerebral hemispheres, imparting different control characteristics to each arm. This has been shown across a range of movements, including horizontal and vertical reaching movements, turn and reach movements, overhand throwing, and through studies of adaptation to novel force environments and to novel visuomotor distortions.

In conclusion, handedness results from the hybridization of predictive and impedance control mechanisms, which have become specialized to different hemispheres. The integration of both control mechanisms into unimanual limb movements ensures both optimality of movement and robustness against unpredictable mechanical conditions. Rogers and colleagues have provided evidence that hemispheric specialization allows for efficient performance of potentially competing neural processes, which emphasizes the importance of lateralization in optimal and adaptive behavior. This view of lateralization provides a fundamental explanation of the motor control mechanisms that result in the emergence of motor performance asymmetries.

While the majority of the studies cited in this paper addressed right-handed individuals, similar findings have also been shown for left handers (Przybyla et al., 2012), suggesting that both expressions of handedness might reflect the same but mirror imaged organization. However, it should be stressed that left-handers often show more symmetric motor behavior, and the extent rather than the direction, of handedness might represent very different neural phenomenon. Because lateralization appears to reflect an optimization process, lack of such lateralization should result in poor integration of predictive with impedance processes for movement control. This should lead to less effective prediction of limb dynamics and lower ability to stabilize against unpredicted perturbations. Such incoordination might be related to fact that children with developmental coordination disorder tend to show lower laterality indices (Hill and Bishop, 1998). However, it is also possible in some individuals that symmetry in behavior could be associated with greater function, as well as greater neural lateralization. In fact, it has been shown that when individuals suffer an amputation of their dominant right arm, they learn to use the previously non-dominant arm as their dominant controller. After years of practice, the non-dominant left arm functions comparably with age matched subjects' dominant arms. This improvement in function of the non-dominant arm is associated with greater activation of ipsilateral cortex, indicating that practice using the non-dominant arm did not cause the nervous system to become symmetric, but rather led to greater access of the lateralized neural system during movement control (Philip and Frey, 2014). This suggests a plasticity in the control system that could allow greater symmetry of function through practice. Thus, it is likely that symmetry in motor performance and preference may represent either optimization of a lateralized neural system, or lack of neural lateralization, which would likely lead to deficiencies in coordination. While this proposition is highly speculative, it provides predictions that can be directly tested through empirical research methods.

## Funding

This work was supported by #R01 HD059783 from the National Institutes of Health (NIH): NICHD. The funders had no role in study design, data collection and analysis, decision to publish, or preparation of the manuscript.

### Conflict of interest statement

The author declares that the research was conducted in the absence of any commercial or financial relationships that could be construed as a potential conflict of interest.
